# Investigating heredity in cutaneous T-cell lymphoma in a unique cohort of Danish twins

**DOI:** 10.1038/bcj.2016.128

**Published:** 2017-01-20

**Authors:** N Odum, L M Lindahl, M Wod, T Krejsgaard, A Skytthe, A Woetmann, L Iversen, K Christensen

**Affiliations:** 1 Department of Immunology and Microbiology, University of Copenhagen, Copenhagen, Denmark; 2 Department of Dermatology, Aarhus University Hospital, Aarhus, Denmark; 3 The Danish Twin Registry, Institute of Public Health, University of Southern Denmark, Odense, Denmark

Cutaneous T-cell lymphomas (CTCLs) are uncommon but potentially fatal malignancies. The most prevalent clinical forms of CTCL are mycosis fungoides (MF) and the more aggressive leukemic variant, Sézary syndrome (SS).^1, 2^ Although the etiology is largely unknown, some lines of evidence indicate that genetic factors and heredity play a role in CTCL. Thus, independent studies reported on strong linkage disequilibrium between MF/SS and specific HLA-class II allotypes in Caucasians and Ashkenazi, indicating the existence of a significant genetic predisposition to CTCL.^3, 4^ Moreover, examples of CTCL occurring conjointly in monozygotic twin pairs have been reported^5, 6^ further suggesting a possible relevance of genetic factors in the CTCL etiology. However, genetic studies in multi-generation families and larger cohorts of twins have never been conducted. Accordingly, we have taken advantage of the Danish Twin Register and other nationwide population-based registers, to study heritability, predisposition to infectious diseases, comorbidity, hospitalizations and mortality for a 30+-year period in a cohort of 42 twins with CTCL (case twins) and their 42 co-twins, 420 age- and sex-matched twin controls (case controls) and their 420 co-twin controls. The 42 twin pairs comprised 13 monozygotic and 27 dizygotic twin pairs, whereas two twin pairs were of unknown zygosity. Patient characteristics of the CTCL case twin cohort are shown in Supplementary Table 1. Female–male ratio was 1:1.8 and the average age at time of the CTCL diagnosis was 53 years (range: 5–85 years) showing that case twins with CTCL did not differ in terms of age at onset and female–male ratio from what has been described in other cohorts of Caucasian patients with CTCL.^7, 8, 9^ Likewise, the mortality was 2.7-fold increased in the case twins compared with the case–controls, adjusted HR 2.65 (95% confidence interval (CI) 1.66–4.24) (Table 1), further indicating that the CTCL cases represented a typical CTCL cohort.^9^ Surprisingly, all twin pairs were discordant for CTCL, that is, none of the co-twins were diagnosed with CTCL. Importantly, all co-twins were monitored from birth until 1 June 2015 or death and none were lost to follow-up. On average, the co-twins that were alive at the time of case diagnosis were monitored for 20 years (range: 3–40 years) after the case twins were initially diagnosed with CTCL. As none of the co-twins were diagnosed with CTCL within a period of minimum 3 years and up to a maximum of 40 years after the corresponding case twin was diagnosed with CTCL, our findings indicated that the complete absence of CTCL in the co-twins was not a result of a short observation time.

Although none of the co-twins developed CTCL, we examined whether they were diagnosed with other hematological malignancies or had an increased frequency of cancer in general. Importantly, none of the co-twins were diagnosed with non-Hodgkin- or Hodgkin lymphomas (Table 2, upper part). Moreover, the frequency of breast cancer, cancers in the respiratory organs and other malignancies was similar among co-twins and co-twin controls (Table 2, upper part, second column versus forth column and data not shown) indicating that the risk of lymphoma and other cancers in co-twins was similar to that of the control population. The frequency of cancer other than lymphoma was comparable in case twins and co-twins (Table 2, upper part, first versus second column). As CTCL patients have an increased risk of infectious diseases such as pneumonia and sepsis (reviewed in Willerslev-Olsen *et al.*^10^), we compared the frequency of infectious diseases in case- and co-twins and the corresponding control cohorts. As expected,^11, 12^ the frequency of pneumonia and sepsis was significantly higher for the CTCL cases (36% and 17%, respectively) than for the case–controls (17% and 4%, respectively, Table 2, upper part), supporting the notion that CTCL patients carry an increased risk of infections due to an impaired immune defense (reviewed in Girardi *et al.*^1^ and Willerslev-Olsen *et al.*^10^). Restricting the analysis to monozygotic twins showed the same picture, that is, a higher frequency of pneumonia and sepsis among CTCL cases than case–controls (Table 2, lower part). In contrast, the frequency of pneumonia and sepsis in co-twins was 14% and 2%, respectively, which was similar to the frequency seen in co-twin controls (12% and 5%, respectively, Table 2, upper part, column two versus column four). The frequency in co-twins of other infectious diseases was also similar to the frequency in the controls (data not shown) indicating that co-twins—unlike their CTCL case twins—did not display an increased risk of retracting infectious diseases and chronic infections. In support, the frequency of hospitalization was not increased in the co-twins when compared with the co-twin control population (Table 2, upper part, column two versus column four). The frequency of common diseases such as ischemic heart disease, hypertension and chronic obstructive pulmonary disease was also similar in co-twins and co-twin controls (Table 2, upper part, column two versus column four), indicating that the overall morbidity and disease spectrum in co-twins was very similar to that seen in the control population and distinctly different from their CTCL case twins. Indeed, we found no difference in mortality between co-twins and co-twin controls, adjusted HR 1.08 (95% CI 0.65–1.82) (Table 1), whereas the mortality was increased in the CTCL cases compared with their co-twins, adjusted HR 1.91 (95% CI 0.81–4.50) (Table 1). Causes of death were predominated by malignant diseases in the CTCL case cohort, accounting for 67% of all deaths, whereas malignancy accounted for 31%, 20% and 29% of all deaths in the co-twin, case–control and co-twin control cohorts, respectively (data not shown), further supporting that the CTCL cases represented a typical CTCL cohort whereas the co-twins were similar to the background population in terms of morbidity and mortality.

As mentioned above, the finding that none of the co-twins were diagnosed with CTCL was surprising given the previous case reports of CTCL concordance in two twin pairs^5, 6^ and no reports of discordant twins. However, the result was very clear as none of the co-twins were diagnosed with CTCL or other non-Hodgkin lymphomas. Likewise, none of the co-twins were assigned other differential cancer diagnosis that could have masked an underlying CTCL. Thus, we found no indications of CTCL being misdiagnosed or overlooked in any of the co-twins. Indeed, if CTCL had been overlooked or misdiagnosed in the co-twins, it would have been expected that affected co-twins would have displayed the typical pattern of comorbidity seen in case-twins, but they did not. Importantly, all co-twins were followed for 20 years (range: 3–40 years) after the corresponding case twin was first diagnosed with CTCL and until termination of the study period (1 January 2015) or death, indicating that the absence of CTCL in co-twins was not a result of an insufficient (too short) observation time. Indeed, in some cases co-twins were followed for 35–40 years without developing the disease. Moreover, none of the co-twins were lost-to-follow-up due to other unrelated causes such as immigration or loss of health insurance (as health care are free of charge). Taken together, our findings indicate the difference between case- and co-twins was real and reflected a true difference in CTCL morbidity and comorbidity between the groups. Thus, it may be concluded that there was no familial aggregation of CTCL in the present cohort of Danish twin pairs.

Another interesting issue in the present study was whether or not susceptibility to infectious diseases was an independent feature or linked to the cancer itself. To address whether case twins displayed a pre-disposition for bacterial infections, we examined whether bacterial infection was diagnosed before or after the date the cancer was first diagnosed. Only one patient was diagnosed with an infectious disease before the cancer was diagnosed and one patient was simultaneously diagnosed with cancer and bacterial infection, whereas the rest were diagnosed after the cancer was first diagnosed (data not shown). The present findings that co-twins—unlike case twins—did not display an enhanced frequency of infectious diseases when compared with the control populations were important as they showed that there was no evidence of familial risk in relation to susceptibility to contract serious infectious diseases in the present cohort of Danish twin pairs. Although our data do not exclude the possibility that susceptibility to infections is a primary etiological factor, they strongly support the notion that a high risk of severe infections in CTCL patients is a secondary event to cancer development.^10, 13^

In conclusion, we found discordance for CTCL in the present cohort of twin pairs. On the basis of clinical characteristics, morbidity, hospitalization and mortality the cohort of CTCL cases represented a typical CTCL patient cohort, whereas the co-cases represented the general population. Thus, our nationwide twin study which included all hospitalizations for a 30+-year period was not able to detect any familial aggregation of CTCL and CTCL-associated susceptibility to severe infectious diseases. As the first CTCL study in a cohort of twin pairs, the present findings may therefore have important future implications for genetic counseling and our understanding of the etiology of CTCL.

## Figures and Tables

**Table 1:** Mortality in the CTCL cases, co-twins, case–controls and co-twin controls

*Mortality*	n	*Deaths*	*HR crude (95% CI)*	*HR adjusted*^a^ *(95% CI)*
Case versus case–control (ref.)	461	144	2.65 (1.66–4.22)	2.65 (1.66–4.24)
Case versus co-twin (ref.)	70	23	1.90 (0.81–4.48)	1.91 (0.81–4.50)
Co-twin versus co-twin control	449	160	1.08 (0.64–1.81)	1.08 (0.65–1.82)

Abbreviations: ref., reference.

a Adjusted for sex.

**Table 2:** Malignancies, other comorbidities and causes of death in the CTCL cases, co-twins, case–controls and co-twin controls

	*Case twins* n *(%)*		*Co-twin* n *(%)*		*Case–control* n *(%)*		*Co-twin control* n *(%)*	
*Cancer*								
Respiratory organs	3	(7)	2	(5)	13	(3)	12	(3)
Breast	1	(2)	1	(2)	10	(2)	6	(1)
Hodgkin lymphoma	1	(2)	0	–	0	–	0	–
Lymphoma incl. CTCL	42	(100)	0	–	1	–	0	–

*Other comorbidities*								
Ischemic heart disease	7	(17)	8	(19)	59	(14)	46	(11)
Hypertension	7	(17)	4	(10)	62	(15)	52	(12)
COPD	8	(19)	4	(10)	32	(8)	34	(8)
Pneumonia	15	(36)	6	(14)	70	(17)	52	(12)
Sepsis	7	(17)	1	(2)	17	(4)	22	(5)
No hospitalization	1	(2)	3	(7)	46	(11)	20	(5)
								
*Causes of death*								
Malignancy	14	(67)	5	(31)	24	(20)	42	(29)
Respiratory diseases	1	(5)	0	–	11	(9)	12	(8)
Heart diseases	0	–	1	(6)	21	(17)	15	(10)
Digestive organ diseases	0	–	3	(19)	2	(2)	9	(6)
Other causes of death	6	(29)	7	(44)	65	(63)	66	(46)
All deaths	21	(50)	16	(38)	123	(29)	144	(34)

Abbreviations: CTCL, cutaneous T-cell lymphoma; COPD, chronic obstructive pulmonary disease.
