# Supplementary material for: Investigating heredity in cutaneous T-cell lymphoma in a unique cohort of Danish twins
Source: Blood Cancer J. 2017 Jan 20;7(1):e517–. doi: 10.1038/bcj.2016.128 (PMC5301035; doi:10.1038/bcj.2016.128)
Supplement: Supplementary Information [file bcj2016128x1.docx]

Supplementary Tables:

Supplementary Table 1. Clinical characteristics of the CTCL twin cohort

|  | CTCL twin cohort (%) |
| --- | --- |
| N | 42 |
| Sex |  |
| Females | 15 (36) |
| Males | 27 (64) |
| Zygosity |  |
| MZ | 13 (31) |
| DZ | 27 (64) |
| SS | 14 (33) |
| OS | 13 (31) |
| UZ | 2 (5) |

Abbreviations: CTCL, cutaneous T-cell lymphoma; MZ, monozygotic; DZ, dizygotic; SS, same sex; OS, opposite sex; and UZ, unknown sex.
